# Short-term outcomes of robotic eTEP versus TAPP for ventral hernia repair: insights from a propensity-matched cohort

**DOI:** 10.1007/s11701-025-02439-6

**Published:** 2025-06-14

**Authors:** Fadl Alfarawan, Baha Aldeen Faraj Ali Qaneer, Anna Vincke, Maximilian Bockhorn, Nader El-Sourani

**Affiliations:** 1 Universitätsklinik für Allgemein – und Viszeralchirurgie, Carl von Ossietzky Universität Oldenburg, Klinikum Oldenburg AöR, Rahel-Strauss-Str. 10, 26133 Oldenburg, Germany; 2 Carl von Ossietzky Universität Oldenburg, Fakultät für Medizin und Gesundheitswissenschaften, Ammerländer Heerstraße 114-118, 26129 Oldenburg, Germany; 3 Klinik für Allgemein-, Viszeral – und Transplantationschirurgie, Universitätsklinikum Münster, Waldeyerstraße 1, 48149 Munster, Germany

**Keywords:** eTEP, TAPP, rVHR, Robotic surgery, Ventral hernia, Retromuscular repair

## Abstract

Robotic ventral hernia repair (rVHR) is an advanced form of minimally invasive surgery that offers enhanced precision, reduced complications, and faster recovery time. However, direct comparisons between enhanced-view totally extraperitoneal (eTEP) and transabdominal preperitoneal (TAPP) approaches remain limited. This study aimed to compare the safety and clinical outcomes of eTEP and TAPP in rVHR. In this retrospective cohort study, 117 patients underwent rVHR (82 eTEP, 35 TAPP) between 2023 and 2024. Propensity score matching (PSM) (1:1) balanced baseline characteristics, resulting in 33 patients per group. Patient demographics, operative data, and postoperative outcomes were collected from electronic medical records. Statistical analyses were conducted using SPSS, with statistical significance defined as *p* < 0.05. Following matching, eTEP demonstrated significantly longer operative times (median 115 vs. 83 min, *p* = 0.004) and larger mesh sizes (420 cm^2^ vs. 195 cm^2^, *p* = 0.001). Surgical drains were more frequently used in eTEP (48.4% vs. 3%, *p* = 0.001). Postoperative outcomes, length of hospital stay, and pain scores did not differ significantly between the groups. Surgical site occurrences (SSOs) showed no significant difference between groups (18.1% eTEP vs. 9% TAPP, *p* = 0.475). Both eTEP and TAPP are safe and effective robotic approaches for ventral hernia repair with comparable clinical outcomes. The longer operative time and larger mesh size in eTEP suggests its preferential use in more complex hernia cases requiring detailed anatomical reconstruction.

## Introduction

Ventral hernias are a common clinical condition that, if left untreated, can lead to serious complications such as incarceration or strangulation [1–3]. Studies indicate that ventral hernia repairs (VHRs) account for approximately one-third of all hernia procedures in the United States, with two-thirds involving primary ventral hernias and one-third comprising incisional hernias [4]. Historically, ventral hernias were treated using the open surgical approach. However, the advent of minimally invasive techniques marked a paradigm shift, beginning with the laparoscopic method introduced by LeBlanc and Booth in 1993 [5]. Laparoscopic repair offers clear advantages over the open technique, including lower complication and recurrence rates, as well as shorter recovery times [6]. Despite these benefits, laparoscopic VHR is not without limitations. Complications such as mesh erosion, fistula formation, and intraperitoneal adhesions have been reported, prompting a shift toward more advanced robotic-assisted approaches [7–9].

In 2015, the robotic-assisted transabdominal preperitoneal (r-TAPP) approach was introduced for VHR [10]. Three years later, Belyansky et al. described the enhanced-view totally extraperitoneal (eTEP) technique as a novel minimally invasive method for VHR [11]. Both TAPP and eTEP prioritize anatomical mesh placement in the extraperitoneal space, thereby minimizing complications associated with intraperitoneal contact. A recent meta-analysis confirmed that the TAPP approach reduces hospital stays and early postoperative complications compared to the intraperitoneal onlay mesh (IPOM) technique [12]. Similarly, eTEP positions the mesh within the retrorectus space, avoiding intraperitoneal contact and reducing the risk of adhesions. Wieland et al. reported favorable outcomes with eTEP over IPOM, including shorter hospital stays and lower complication rates [13]. When performed robotically, both approaches offer distinct advantages, including enhanced precision during dissection, more efficient component separation, and improved mesh fixation [14]. A recent propensity score-matched study by Al-Salemi et al. supported these findings, demonstrating that robotic eTEP achieved comparable operative times to laparoscopic eTEP, despite being used for significantly larger hernia defects and requiring larger mesh sizes. Furthermore, robotic eTEP was associated with a significantly shorter hospital stay without an increase in complication rates, underscoring its value in complex hernia repairs [15].

Despite growing adoption of robotic techniques, direct compiarsons between robotic eTEP and TAPP approaches remain limited and inconclusive. Many existing studies suffer from small sample sizes or lack robust methodologies [16, 17].

This study aimed to address these limitations by comparing intraoperative and short-term postoperative outcomes between robotic eTEP and TAPP approaches in a propensity-matched cohort. By ensuring balanced baseline characteristics, this study sought to minimize bias and provide more robust comparative data on the efficacy and safety of these two robotic-assisted approaches for VHR.

## Materials and methods

### Study design

This study is a retrospective cohort analysis based on a prospectively maintained database. It included patients who underwent rVHR using either eTEP or TAPP between 2023 and 2024. The study was performed in line with the principles of the Declaration of Helsinki. Institutional Review Board approval (IRB) was obtained by the Ethics Committee of the Carl von Ossietzky University Oldenburg (2024-096), and all patient data were extracted from electronic medical records.

### Patient selection

**Fig. 1:**
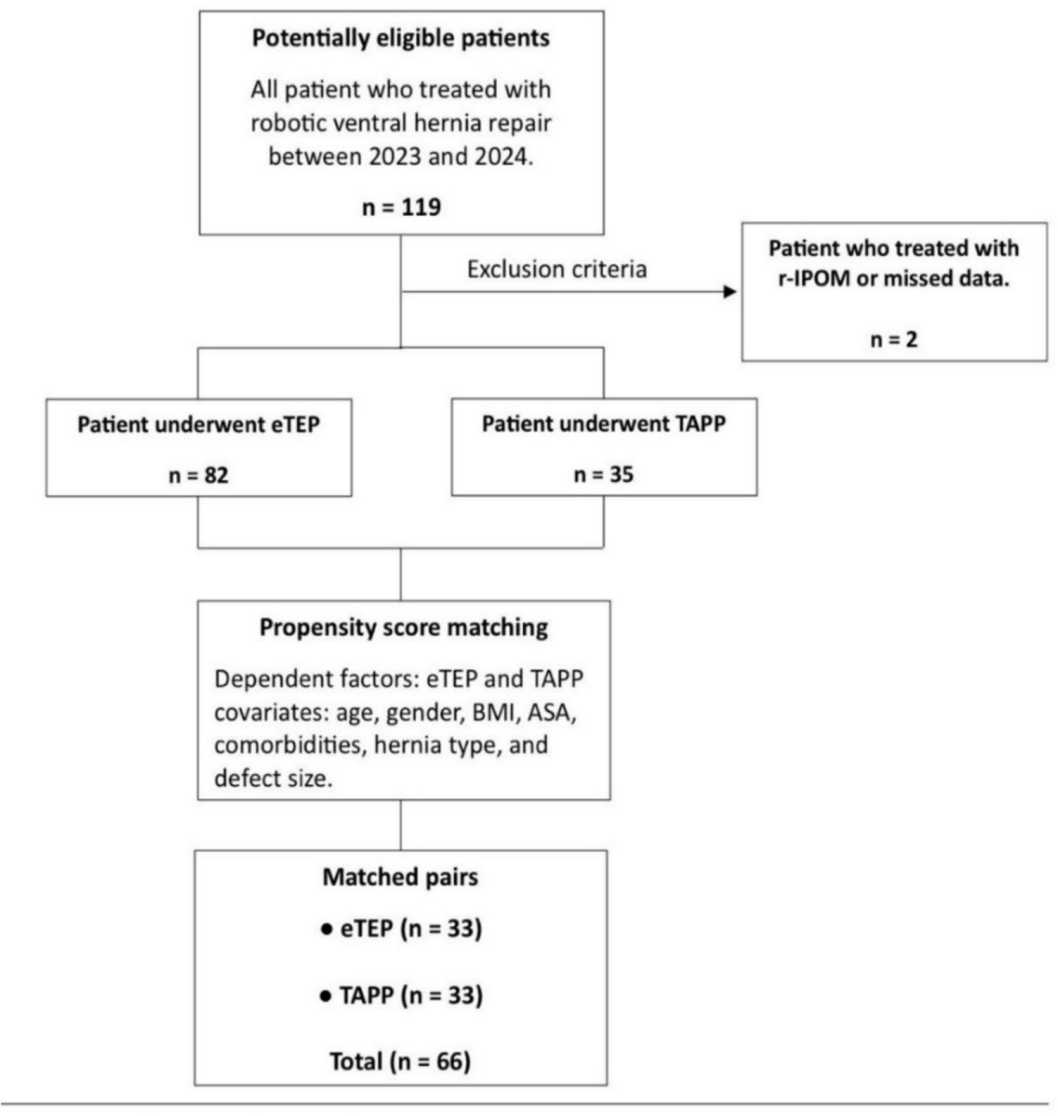
Flowchart illustrating patient selection

From the total cohort of rVHRs, only patients who underwent eTEP, TAPP, or concomitant adjunctive transversus abdominis release (TAR) were included. Patients who underwent robotic IPOM were excluded. A flowchart illustrating the patient selection process is shown in Fig. 1.

### Data collection

Patient data were retrieved from electronic medical records and categorized into three main groups: demographics, operative variables, and postoperative outcomes.

Demographics included gender, age, American Society of Anesthesiologists (ASA) classification score, body mass index (BMI), and comorbidities, including chronic obstructive pulmonary disease (COPD)/asthma, cardiovascular diseases, diabetes mellitus, anticoagulant use, nicotine abuse, and hernia characteristics, such as hernia type, location, and defect size. Operative variables encopmassed the surgical approach, operative time, use of adjunctive TAR, mesh size, intraoperative complications, conversion to open surgery, and the necessity for postoperative drainage. Postoperative outcomes were assessed using various parameters. Pain was measured using the visual analog scale (VAS), in addition, length of hospital stay, complication rates, recurrence rates, and postoperative complications using the Clavien–Dindo classification system [18], surgical site occurrences (SSOs) and surgical site infections (SSIs) were recorded.

### Data analysis

Statistical analysis was performed using SPSS version 28. Continuous variables were presented as means with standard deviations or medians with interquartile ranges, depending on data distribution. Categorical variables were reported as frequencies with percentages. Group comparisons were conducted using independent t-tests for normally distributed continuous variables, Mann–Whitney U tests for non-normally distributed continuous variables, and chi-square or Fisher’s exact tests for categorical variables. A *p*-value of < 0.05 was considered statistically significant.

To minimize selection bias, PSM was performed using the nearest neighbor method with a caliper of 0.1 in a 1:1 ratio, resulting in 33 patients per group.

Matching criteria included age, gender, BMI, ASA score, hernia type and size, and comorbidities to ensure comparable baseline characteristics between the eTEP and TAPP groups.

## Results

### Patient demographics

A total of 117 patients were included in the study, with 82 undergoing the eTEP approach and 35 undergoing the TAPP approach. To enable a balanced comparison, we performed propensity score matching using nearest neighbor matching (1:1 ratio, without replacement, caliper = 0.01). The standardized mean difference (SMD) after matching was 0.0092 (< 0.1), indicating excellent balance between groups, whereas the SMD prior to matching was 0.81505. In total, 20 cases were matched exactly, and 12 cases were matched nearly exactly (± 0.01). PSM resulted in a matched cohort of 66 patients―33 in each group. A table comparing the SMD values before and after matching is provided in the appendix.

Gender distribution was comparable between groups, with males comprising 69.7% of the eTEP group and 60.6% of the TAPP group (*p* = 0.438). The mean age was slightly higher in the eTEP group (58 ± 12 years) compared to the TAPP group (54 ± 15 years, *p* = 0.266). The median BMI was similar between groups [27 (IQR 25–32.5) for eTEP vs. 30 (IQR 26–34.5) for TAPP, *p* = 0.298]. Most patients were classified as ASA 2 (60.6% in eTEP and 66.6% in TAPP, *p* = 0.872).

The most common hernia type was incisional hernia, observed in 54.5% of eTEP patients and 69.7% of TAPP patients (*p* = 0.428). Umbilical hernias were present in 42.4% of eTEP cases and 27.3% of TAPP cases, while epigastric hernias were seen in 3% of eTEP patients and 3% of TAPP patients. Median defect size did not significantly differ between groups (21 for eTEP and 12 for TAPP, *p* = 0.436). There were also no significant differences between the two groups in terms of hernia width according to the European Hernia Society (EHS) classification. A table is provided in the appendix [19].

Comorbidities were similarly distributed. Cardiovascular disease was present in 48.4% of the eTEP group and 45.4% of the TAPP group (*p* = 0.805). Rates of diabetes (12.1% vs. 15.1%, *p* = 1.000), COPD/asthma (15.1% vs. 15.1%, *p* = 1.000), smoking history (27.3% vs. 30.3%, *p* = 0.786), and anticoagulant use (12.1% vs. 9%, *p* = 1.000) were also comparable. Baseline characteristics before and after matching are summarized in Table 1.

**Table 1:** Baseline characteristics before and after matching

	Unmatched group			Matched group		
	eTEP n = 82	TAPP n = 35	*p*	eTEP n = 33	TAPP n = 33	*p*
Age (years), mean ± SD	56 ± 14	52 ± 16	0.266	58 ± 12	54 ± 15	0.254
Sex, male, n (%)	49 (59.8%)	22 (62.9%)	0.753	23 (69.7%)	20 (60.6%)	0.438
BMI (kg/m^2^), median (IQR)	29 (9)	30 (10)	0.561	27 (7.5)	30 (8.5)	0.298
ASA score						
1, n (%)	10 (12.2%)	5 (14.3%)	0.581	5 (15.1%)	4 (12.1%)	0.872
2, n (%)	48 (58.5%)	23 (65.7%)		20 (60.6%)	22 (66.6%)	
3, n (%)	24 (29.3%)	7 (20%)		8 (24.2%)	7 (21.2%)	
Risk factors, n (%)						
COPD/asthma	16 (19.5%)	5 (14.3%)	0.500	5 (15.1%)	5 (15.1%)	1.000
DM	9 (11%)	5 (14.3%)	0.756	4 (12.1%)	5 (15.1%)	1.000
Cardiovascular diseases	35 (42.7%)	16 (45.7%)	0.762	16 (48.4%)	15 (45.4%)	0.805
Liver cirrhosis	1 (1.2%)	0	1.000	–	–	–
Smoking	22 (26.8)	10 (28.6%)	1.000	9 (27.3%)	10 (30.3%)	0.786
Anticoagulant use	8 (9.8%)	3 (8.6%)	0.841	4 (12.1%)	3 (9%)	1.000
Defect size (cm^2^), median (IQR)	33.5 (72)	9 (29)	0.013	21 (71)	12 (29)	0.436
Hernia type, n (%)			0.111			0.428
Umbilical	17 (20.7%)	11 (31.4%)		14 (42.4%)	9 (27.3%)	
Epigastric	5 (6.1%)	1 (2.9%)		1 (3%)	1 (3%)	
Incisional	47 (57.3%)	23 (65.7%)		18 (54.5%)	23 (69.7%)	
Umbilical/epigastric	12 (14.6%)	0 (0%)				
Incisional/parastomal	1 (1.2%)	0 (0%)				

### Intraoperative outcomes

The median operative time was significantly longer in the eTEP group (115 min, IQR 51) compared to the TAPP group (83 min IQR 54, *p* = 0.004). The median mesh size used was also significantly larger in the eTEP group (420 cm^2^, IQR 145) than in the TAPP group (195 cm^2^, IQR 150, *p* = 0.001). TAR was performed more frequently in the eTEP group (18.2%) than in the TAPP group (12.1%), although the difference was not statistically significant (*p* = 0.230). The use of surgical drains was significantly higher in the eTEP group (48.4%) compared to the TAPP group (3%, *p* = 0.001). No intraoperative complications or conversions to open surgery were reported in either group, as shown in Table 2.

**Table 2:** Intra-operative variables before and after matching

	Unmatched group			Matched group		
	eTEP n = 82	TAPP n = 35	*p*	eTEP n = 33	TAPP n = 33	*p*
Mesh size, cm (IQR)	450 (225)	180 (150)	0.001	420 (145)	195 (150)	0.001
Transversus abdominis release, n (%)	17 (20.7%)	4 (11.4%)	0.230	6 (18.2%)	4 (12.1%)	0.492
Operative time, min (IQR)	123 (63)	83 (53)	0.001	115 (51)	83 (54)	0.004
Drainage placement, n (%)	40 (48.8%)	1 (2.9%)	0.001	16 (48.4%)	1 (3%)	0.001
Intra-operative complications, n (%)	0 (0%)	0 (0%)	–	0 (0%)	0 (0%)	–
Conversion, n (%)	0 (0%)	0 (0%)	–	0 (0%)	0 (0%)	–

### Postoperative outcomes

Postoperative complications were minimal, occurring in one patient in the eTEP group (3%), while none were observed in the TAPP group (*p* = 1.000). The median length of hospital stay was 2 days for both groups (*p* = 0.102). Pain levels, assessed using the VAS, showed no significant differences between the groups on postoperative days 1 (*p* = 0.530), 2 (*p* = 0.150), and 3 (*p* = 0.366). No SSIs were reported in either group, SSOs were occured (18.1%) in the eTEP group compared to the TAPP group (9%), which the difference was not statistically significant (*p* = 0.475). The majority of complications were seromas, most of which were managed on an outpatient basis, while some patients were discharged with drainage for continued management at home. Despite the difference in SSO rates, both approaches demonstrated an overall manageable complication profile with effective postoperative care. According to the Clavien–Dindo classification, minor complications were observed at low rates in both groups, including Grade I (0% in eTEP vs. 3% in TAPP), Grade II (6% in eTEP vs. 2% in TAPP), and Grade III (3% in eTEP vs. 0% in TAPP), with no statistically significant difference (*p* = 0.949). All postoperative variables are summarized in Table 3.

**Table 3:** Postoperative variables before and after matching

	Unmatched group			Matched group		
	eTEP n = 82	TAPP n = 35	*p*	eTEP n = 33	TAPP n = 33	*P*
Postoperative complications, n (%)	3 (3.7%)	0 (0%)	0.553	1 (3%)	0 (0%)	1.000
Clavien Dindo, n (%)			0.680			0.949
I	3 (3.6%)	1 (2.9%)		0 (0%)	1 (3%)	
II	4 (4.9%)	2 (5.7%)		2 (6%)	2 (6%)	
III	2 (2.4%)	0 (0%)		1 (3%)	0 (0%)	
Admission period	2 (0 IQR)	2 (0 IQR)	0.155	2 (0 IQR)	2 (0 IQR)	0.102
VAS						
Postoperative day 1	2 (3 IQR)	2 (2 IQR)	0.939	1 (3 IQR)	2 (2 IQR)	0.530
Postoperative day 2	1 (2 IQR)	1 (3 IQR)	0.132	1 (2 IQR)	1 (3 IQR)	0.150
Postoperative day 3	1 (2 IQR)	2 (4 IQR)	0.261	1 (1 IQR)	2 (4 IQR)	0.366
SSI, n (%)	1 (1.2%)	0 (0%)	1.000	0 (0%)	0 (0%)	–
SSO, n (%)	9 (10.1%)	3 (8.6%)	1.000	6 (18.1%)	3 (9%)	0.475

## Discussion

This study compared intraoperative and short-term postoperative outcomes between robotic eTEP and TAPP approaches for ventral hernia repair. We found that while eTEP was associated with longer operative times and the use of larger mesh, both approaches demonstrated comparable safety profiles and postoperative outcomes.

Our findings showed that the eTEP approach had significantly longer operative times compared to TAPP (median 115 vs. 83 min, *p* = 0.004). This is consistent with results from Kudsi et al. (17), and is likely attributable to the more complex anatomical dissection required in eTEP. Specifically, retromuscular dissection and posterior component separation, often necessary in eTEP, contribute to extended procedural time. Additionally, the technical demands of preperitoneal space creation and the need for meticulous hemostasis further prolong the procedure. However, our findings differ from Pini et al., who reported longer operative times in TAPP compared to eTEP [16]. These discrepancies may reflect variations in surgeon experience, case complexity, and learning curves, emphasizing the need for further study into factors influencing operative efficiency in rVHR.

We observed significantly more frequent use of surgical drains in the eTEP group compared to TAPP (48.4% vs. 3%, *p* = 0.001), a finding that contrasts with Pini et al., who found no significant difference in drain placement (3.1% vs. 9.5%, *p* = 0.329) [16]. This variation may be due to differences in surgeon preference, perceived risk of seroma after retromuscular dissection, or institutional protocols. Notably, Wilters et al. reported no significant differences in postoperative outcomes with or without drain placement in eTEP, suggesting that this practice may be more precautionary than evidence-based [20]. These inconsistencies highlight the lack of standardized guidance on drain usage in robotic VHR.

Our data showed that eTEP utilized significantly larger mesh sizes compared to TAPP (420 cm^2^ vs. 195 cm^2^, *p* = 0.001), in line with the findings of Kudsi et al., who noted that eTEP accommodates larger hernia defects due to its access to the retromuscular plane [21].

Larger mesh placement may enhance durability and reduce recurrence risk by achieving broader overlap. Furthermore, we address the discrepancy observed between defect and mesh sizes. The larger mesh sizes in the eTEP group are primarily attributable to the nature of the technique, which facilitates―and often necessitates―a more extensive retrorectus and preperitoneal dissection. This broader dissection allows for greater mesh overlap. In contrast, TAPP repairs are typically constrained by the intraperitoneal space and the dimensions of the peritoneal flap, often limiting the feasible mesh size [17].

We analyzed the width of hernia defects using the European Hernia Society (EHS) classification system. Although there were numerically more W3 defects in the eTEP group, the difference in transverse diameter compared to the TAPP group did not reach statistical significance (*p* = 0.086). This trend, however, may reflect a selection bias favoring more complex or larger hernias in the eTEP cohort. Such a tendency is consistent with previous findings by Kudsi et al. [21], who demonstrated that the eTEP technique is particularly suitable for managing larger defects due to its capacity to access and utilize the retromuscular plane. This anatomical advantage not only facilitates broader mesh placement and greater overlap but also enables the surgeon to address more challenging hernia configurations.

There were no significant differences in hospital stay duration (*p* = 0.102) or postoperative pain (VAS scores on Days 1–3: *p* = 0.530, 0.150, and 0.234, respectively) between groups, consistent with prior reports [21]. These findings suggest that both approaches provide similar short-term recovery profiles.

Consistent with the literature, we found no statistically significant difference in SSO rates between groups (18.1% for eTEP vs. 9% for TAPP, *p* = 0.475). Previous studies have similarly reported low SSO rates for both approaches [21, 22]. Postoperative complications were minimal, with only one complication (fistula and mesh migration) in the eTEP group and none in TAPP, mirroring the findings by Pini et al. [16]. Additionally, the extraperitoneal nature of eTEP may reduce adhesion risk, as suggested by Farmer et al. [23].

Both robotic eTEP and TAPP are effective and safe approaches for ventral hernia repair, offering comparable short-term outcomes. While eTEP allows for enhanced anatomical reconstruction and larger mesh use―ideal for complex hernias―it comes at the cost of longer operative time. TAPP remains a suitable option for less complex cases requiring shorter procedural duration. Future multicenter, prospective studies with long-term follow-up and incorporation of patient-reported outcomes are essential to optimize approach selection in robotic ventral hernia repair.

## Limitation

This study’s strengths include the use of propensity score matching to balance baseline characteristics and reduce selection bias, as well as detailed intraoperative and postoperative data collection. However, several limitations must be acknowledged. The retrospective design introduces inherent bias, and the short follow-up period limits assessment of recurrence and long-term complications. As a single-center study, the generalizability of our findings is constrained by institutional and surgeon-specific factors. The relatively small sample size, particularly after matching, may limit the ability to detect smaller but clinically meaningful differences between groups.

In addition, while hernia type was included as a covariate in the propensity score model, it was not used as an exact match criterion. In retrospect, we recognize this as a methodological limitation. Our decision was driven by the relatively small sample size, which would have significantly reduced statistical power had exact matching on hernia type been applied. This may have allowed for residual imbalance between groups and could limit the strength of causal inference. Future studies should consider exact matching for key clinical variables such as hernia type to further enhance the robustness and comparability of propensity-matched analyses.

## Conclusion

eTEP offers specific technical and biomechanical advantages, including the potential for retromuscular mesh placement with broad overlap. These benefits come at the cost of increased operative complexity and duration. TAPP, on the other hand, may be more suitable for smaller defects or when shorter operative times are preferred. The choice of technique should therefore be guided by individual patient anatomy, surgeon experience, and institutional capabilities.

## Data Availability

No datasets were generated or analysed during the current study.

## Appendix

### Standardized mean differences (SMD)—before and after matching

**Table:**

Variable	SMD Before	SMD After
Age	0.22	0.28
Gender	0.06	0.19
ASA Score	0.18	0.00
COPD	0.16	0.00
Diabetes	0.09	0.086
Heart-related issue	0.06	0.059
Liver failure	0.091	0.00
Nicotine use	0.044	0.065
Anticoagulant use	0.034	0.03
Hernial gap (cm^2^)	0.39	0.068
Hernia type	0.64	0.28

### Hernia width according to European Hernia Society after Matching

**Table:**

		eTEP		TAPP		*P*
		n	%	n	%	
EHS	W1	14	43.75	19	59.38	0.196
	W2	7	21.88	8	25	1.000
	W3	11	34.38	5	15.63	0.086
